# Mineral Density Volume Gradients in Normal and Diseased Human Tissues

**DOI:** 10.1371/journal.pone.0121611

**Published:** 2015-04-09

**Authors:** Sabra I. Djomehri, Susan Candell, Thomas Case, Alyssa Browning, Grayson W. Marshall, Wenbing Yun, S. H. Lau, Samuel Webb, Sunita P. Ho

**Affiliations:** 1 Division of Biomaterials and Bioengineering, Department of Preventive and Restorative Dental Sciences, University of San Francisco, San Francisco, California, United States of America; 2 Xradia Inc., Pleasanton, California, United States of America; 3 Stanford Synchrotron Radiation Lightsource, Stanford Linear Accelerator Center, Menlo Park, California, United States of America; Brigham and Women's Hospital, Harvard Medical School, UNITED STATES

## Abstract

Clinical computed tomography provides a single mineral density (MD) value for heterogeneous calcified tissues containing early and late stage pathologic formations. The novel aspect of this study is that, it extends current quantitative methods of mapping mineral density gradients to three dimensions, discretizes early and late mineralized stages, identifies elemental distribution in discretized volumes, and correlates measured MD with respective calcium (Ca) to phosphorus (P) and Ca to zinc (Zn) elemental ratios. To accomplish this, MD variations identified using polychromatic radiation from a high resolution micro-computed tomography (micro-CT) benchtop unit were correlated with elemental mapping obtained from a microprobe X-ray fluorescence (XRF) using synchrotron monochromatic radiation. Digital segmentation of tomograms from normal and diseased tissues (N=5 per group; 40-60 year old males) contained significant mineral density variations (enamel: 2820-3095mg/cc, bone: 570-1415mg/cc, cementum: 1240-1340mg/cc, dentin: 1480-1590mg/cc, cementum affected by periodontitis: 1100-1220mg/cc, hypomineralized carious dentin: 345-1450mg/cc, hypermineralized carious dentin: 1815-2740mg/cc, and dental calculus: 1290-1770mg/cc). A plausible linear correlation between segmented MD volumes and elemental ratios within these volumes was established, and Ca/P ratios for dentin (1.49), hypomineralized dentin (0.32-0.46), cementum (1.51), and bone (1.68) were observed. Furthermore, varying Ca/Zn ratios were distinguished in adapted compared to normal tissues, such as in bone (855-2765) and in cementum (595-990), highlighting Zn as an influential element in prompting observed adaptive properties. Hence, results provide insights on mineral density gradients with elemental concentrations and elemental footprints that in turn could aid in elucidating mechanistic processes for pathologic formations.

## Introduction

Connective tissues such as alveolar bone, cementum, dentin, and enamel undergo a concerted hierarchical assembly of proteins and minerals. Alveolar bone that interfaces with a tooth via the periodontal ligament (PDL) is unique, in that, it is structurally and chemically different with age, and in comparison to skeletal bone, cementum, dentin, and enamel. Heterogeneities resulting in mineral densities are influenced by age related physiology and environmental effects[1], and can collectively affect bone quality. This is because the functional units of bone are due to the effect of different rates of cellular events within bone. Cellular events depend also on duration, frequency, and magnitude of external stimuli[2,3]. Hence, it is conceivable that the amount and type of mineral, and resulting mineral density gradients could vary depending on the intensity of stimulus felt by the cells.

Within the periodontal complex, bone does not exist by itself. Alveolar bone is attached to cementum, a mineralized tissue often radiographically identified as an outer layer of the root. Cementum is known to be less mineralized than bone and dentin, and is considered to have a lamellar structure similar to alveolar bone[4]. Secondary cementum covering the apical ends of the tooth root can predominantly go through load-mediated adaptation, transduced by cells to prompt adaptive properties commonly identified as heterogeneous structures with varied chemical compositions. In cementum, the variance in chemical composition is thought to be due to diet and other environmental sources resulting in radiopaque incremental lines[4,5]. Hence it is plausible that cementum can have varied elemental composition and perhaps varied amounts of mineral density which can serve as annual finger-prints for season related diet types within mammals. This implies that environmental influences could manifest into mineral gradients within secondary cementum. While cell-mediated mineralization processes underline the intrinsic nature of cementum, periodontitis-affected cementum and changes in mineral density can lead to the formation of dental calculi, often considered as an ectopic mineral. Supra- and subgingival[6] calculi are thought to arise from supersaturation of ions in saliva and/or bacteria-induced mineralization[7]. Given the disparate ways in which calculi form, it may mix with adapted cementum resulting in its concretion and varying mineral forms[8].

Within a tooth, cementum is chemically and mechanically integrated with dentin via a 100–200 μm cementum dentin interfacial zone[9]. Dentin can be subcategorized into mantel, primary, secondary, and tertiary forms. Mantle dentin is often a 100–200 μm layer around tubular dentin that lacks characteristic dentin tubules and is lower in mineral density, found in both crown and root[7]. However, primary and secondary dentin contain substructures that include peri-, inter, or intra-tubular[10] dentin. Interestingly, with age and disease, the tubules become occluded to minimize tooth related sensitivity and tooth decay[11]. Hence, it is likely that the innate defense mechanism can prompt heterogeneous mixtures of inorganic and organic ratios in dentin, and these changes in turn can be distinguished as mineral gradients.

The formed bioapatite regardless of normal or pathologic in nature is thought to occur due to two broadly classified biomineralization processes: biologically controlled (cell-mediated), and/or biologically induced (physical chemistry)[12,13]. In cell-mediated mineralization processes, the mineralizing volumes on organic matrices of normal tissues are formed over a course of development and functional growth of organisms. Superposition of exogenous biofactors with endogenous events can prompt a change in rate of a biomineralization event which in turn can prompt different mineral phases, types, textures, and amounts per unit volume of a matrix. However, quantity and elemental information can be used as correlative markers to map heterogeneity in mineralized tissues throughout the life span of an organism. The quantity of mineral is often evaluated using a calibrated computed-tomography (CT) bench-top unit where the absorbed x-ray energy is directly proportional to the amount of mineral within a volume of tissue. Complementing mineral density volume information are maps of x-ray fluorescence signals gathered from elements within discrete volumes by using a microprobe.

Micro-computed tomography (micro-CT) is a non-destructive imaging modality in which three-dimensional (3D) mineral volumes can be virtually sectioned and correlated with internal structures. Although micro-CT has been used for a diverse range of qualitative dental and bone studies, technological advances have challenged quantitative investigations[14], such as the distribution of mineral concentration in human enamel and dentin[15,16], skeletal bone[17,18], cementum[19], studies on tooth morphometrics[20], and dental pathology[21,22]. However, the range of micro-CT studies at present have not explored the heterogeneity in mineral density and correlation of mineral patterns to elemental mapping in normal and pathologic tissues within the broader scope of biomineralization. Experimental methods to date that complement CT investigations include the widely used gravimetric analysis, with limited information on local specificity. Other complementary studies that provide spatially resolved elemental mapping include energy dispersive x-ray analysis (EDX), microprobe X-ray fluorescence (XRF) for elemental analysis, X-ray Absorption Near Edge Structure (XANES) while accounting for matrix structure, and mineral density values within volumes of tissues by using higher resolving capacity of x-ray microscopes. This study exploits high resolution microscopy, tomography, and spectroscopy techniques to map heterogeneous mineral density volumes regardless of mineralization processes in alveolar bone, cementum, and dentin that are part of the dentoalveolar complex. Hence, within this section a succinct description about structure, chemical composition about each tissue as investigated by studies to date will be provided.

The tissues, alveolar bone, cementum and dentin are constantly exposed to the conditions of the oral environment, a large bioreactor in which exist several microbioata, changes in pH, temperature, elemental ions, chewing forces and fluid flow. All these factors can affect the organic-to-inorganic ratio within and across tissues, both spatially and temporally. In literature, mineral density ranges exist for normal enamel (2170–3100 mg/cc)[16,23], dentin (1290–1530 mg/cc)[16,23], and skeletal bone (trabecular: 150–1180 mg/cc, cortical: 825–1280 mg/cc)[18,24], while no mineral density range exists for normal cementum or alveolar bone. Studies on tooth pathology show the mineral range of enamel caries lesions (2100–2700 mg/cc)[25], and caries-affected dentin reported as hypomineralized (minimum: 550 mg/cc), hypermineralized (maximum: 2250 mg/cc)[26], as mineral content in volume percent[27], Hounsfield units[28], or qualitatively[29]. However, no mineral density ranges exist in literature for pathologic dentin, cementum, or dental calculus, including no quantitative method which fully exploits 3D micro-CT data. Instead, CT methods in literature continue to represent pathologic tissue volumes as a single value within which information on mineral varieties can exist.

The results of this study provide an extended perspective on mineral density gradients by correlating gradients with concentrations of apatite forming elements: calcium, phosphorus, and zinc. In the literature, ranges exist for the Ca/P ratio of enamel (2.3–2.4)[30], bone (1.63–2.01)[31,32], pathologic bone (1.48–1.55)[32], cementum (1.3–1.97)[30,31], acellular cementum (1.65)[30], dentin (2.1) and hypomineralized dentin (1.9)[33], and dental calculus (0.9–1.7)[34–36]. Although Ca/Zn ratios in various mineral formations in humans have not been investigated, recent studies have observed a correlation between Ca and Zn content, including Zn/Ca ratios in rat bones[37] and in cementum and periodontal disease[38]. A variety of other elements have also been detected in bone[39] and teeth[40], notably Mg, Na, K, Cl, Zn, Cu, Sr, Pb, and Mn. In bone, accumulations of Zn, Sr, and Pb were found in cement lines[41], and distributions of Zn, Cu, Pb, Ni and Hg were found in periodontically affected cementum[38]. Other notable investigations found Zn to be five times higher in cementum over dentin[42,43] and in subgingival calculi over supragingival calculi[44–46]. In this study, digital segmentation of both micro-CT and XRF data will enable correlated mapping of elemental composition (Ca/P, Ca/Zn ratios) within discrete volumes of mineral density (MD) values.

## Materials and Methods

### 2.1. Ethics Statement

All specimens were obtained using self-certification for non-human subjects research as approved by Committee on Human Research (CHR), The Human Research Protection Program at UCSF (http://www.research.ucsf.edu/chr). Institutional review board waived the need for written informed consent from the participants as the data were analyzed anonymously.

### 2.2. Specimen Preparation

Normal and diseased permanent molars extracted from fourteen 40–60 year old male patients were obtained following a dental treatment and as approved by UCSF Committee on Human Research following NIH guidelines. Five mineralized tissues were isolated by sectioning using a low-speed water-cooled diamond saw (Isomet, Buehler, Lake Bluff, IL). 1 mm thick beams of dentin and enamel with an intact dentin enamel junction were prepared. 100–150 μm thick cementum specimens were prepared by coring out dentin using a hand drill and 200–800 grit paper (True Speed 2, Cardinal Dental Laboratory Inc., Walnut Creek, CA). After being removed from molars, 200 μm thick alveolar bone specimens were extracted using a bone cutter. Mineral density variations in diseased dentin specimens containing carious lesions at least over 1 mm radius were evaluated. Diseased cementum was sectioned from the roots of molars that had a measured periodontal attachment loss greater than or equal to 3 mm and with subgingival calculus. Ectopic calcified masses, that is, sub- and supragingival calculus was obtained while scaling and root planing was being performed. All specimens were stored and scanned under wet conditions in Hank's solution (Hank’s Balanced Salt Solution 1x HBSS without Ca & Mg Salts w/Phenol Red, CCFAJ003, UCSF Cell Culture Facility, Media Production Formulations, San Francisco, CA).

### 2.3. Hounsfield Unit (HU) vs. Mineral Density using phantoms

**Fig 1:**
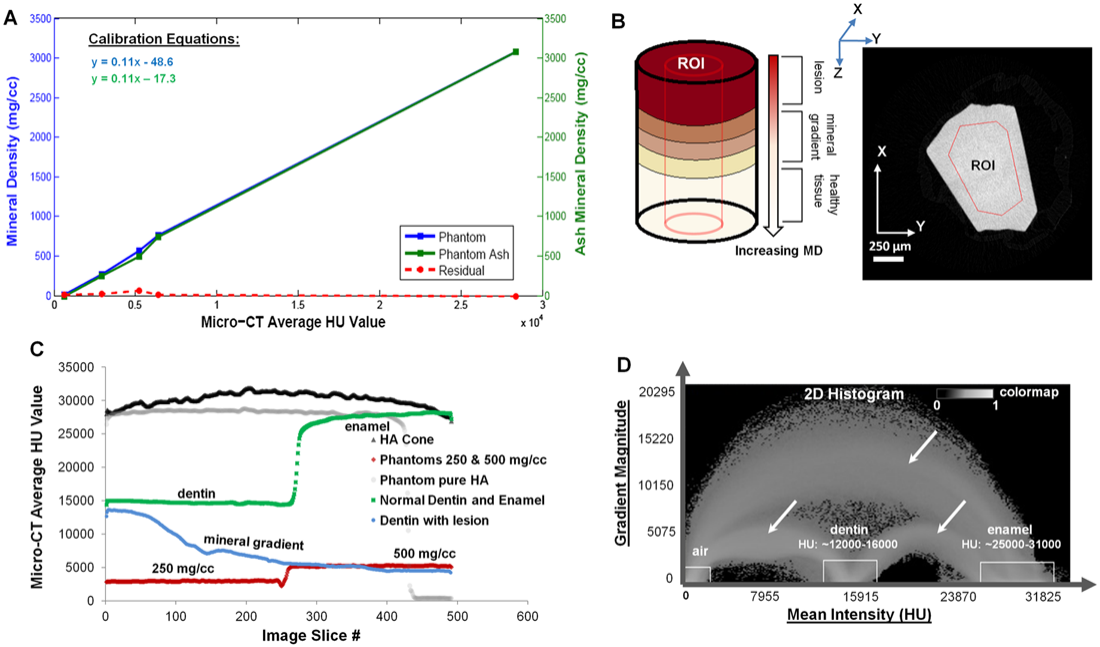
Mineral density calibration. A) Computed mineral density vs. HU calibration curve using phantoms (blue line, left Y-axis) ranging from 0–3080 mg/cc. Ash mineral density of the same calibration phantoms (green line, right Y-axis) is correlated with the computed calibration curve (blue curve), and the difference is plotted (red dashed line). B) Schematic of an experimental specimen and postulated mineral gradients illustrating a virtual x-y slice of a mineralized tissue (dentin) with a region of interest (ROI); C) Average HU (Y axis) distribution corresponding to a ROI (X axis) in each slice for different phantoms and experimental specimens; D) Image segmentation for mineral gradient evaluation using a 2D histogram Avizo Fire 7 illustrates ranges which were used as initial values and the arches (white arrows) represent an interface between two different materials (e.g. dentin and enamel).

An X-ray micro-computed tomography unit (micro-CT, Xradia, Pleasanton, CA) was calibrated using mineral density phantoms of concentrations at 0, 250, 500, 747, 750, 1136, and 3080 mg/cc (CIRS, Norfolk, VA; Ratoc System Engineering, Inc Ltd, Tokyo, Japan; Gammex RMI, Middleton, WI). CT scaling (proprietary, CT Scaling Instructions; Xradia, Inc) was performed first by scanning phantoms under wet conditions and the intensity values were scaled to intensity values corresponding to attenuation of water (1000 Hounsfield (HU)) and air (0 HU)[47]. A 10X magnification was chosen, along with a peak voltage of 40 kVp, an LE #2 source filter, and a beam hardening constant of 4. Calibration analysis is outlined in Fig 1. A linear relationship between mean HU units and mineral density was established by the calibration curve (blue curve, Fig 1A). Calibration phantoms represented the range of mineral density of the various mineralized tissues analyzed in the study. To note, the phantoms (0, 250, 500, 750 mg/cc) underwent gravimetric analysis (green curve, Fig 1A). See 2.7 and 3.1 for further details.

### 2.4. Mineral densities of normal and diseased tissue volumes

Following x-ray scanning of all specimens, reconstructed volumes (using proprietary XMReconstructor software) were segmented based on resolved differences in intensities within the normal and diseased beams (N = 5 per group). Determining mineral density ranges for normal and diseased tissues involved two different processes. For normal specimens, the XMController 7.0, which is the data acquisition software for the micro-CT, was used to upload CT-scaled reconstructed files. From each curve, the mean HU of a tissue was calculated by choosing an ROI, and the calibration curve (see 2.3) was used to convert mean HU to mineral density in mg/cc. A schematic of an ROI is shown in Fig 1B, with a plot of ROIs from various sample data given in Fig 1C. It should be noted that gravimetric analysis was performed only on normal specimens of bone and dentin and not on diseased specimens (see section 2.7).

The reconstructed tomograms were subsequently processed to detect varying mineral density volumes through a segmentation procedure. Digital segmentation was performed by post-processing software (Avizo Fire 7). In brief, steps involved generating a 2D histogram of the intensity gradient magnitude, with a sample histogram of normal dentin and enamel shown in Fig 1D. The concept used to digitally segment volumes of detectable intensity differences depends on the magnitude of intensity and change in magnitude specific to a direction (vector) at a pixel[48]. Loci (white boxes, Fig 1D) representing HU ranges served as initial values to subsequently segment out volumes of varying mineral densities through an iterative process by using the watershed algorithm[49,50]. For further details, see S1 Supplemental Information on digital segmentation.

### 2.5. X-ray imaging of normal and diseased specimens

Specimens were categorized according to type of tissue and disease. The categories were: normal enamel, normal and disease-affected dentin and cementum, calculus, normal bone and adapted bone. A normal or diseased condition was known based on clinical information for each patient. Experimental conditions that were used to scan the specimens (N = 5/group) included a power of 8 W, current of 175 μA, source distance of 26.5 mm, detector distance of 10.2 mm, camera binning 4, exposure time ~3 s, angle sweep from -180° to 180°, and a total number of 750 image projections. The voxel size from the tomograms was 3.84 μm. All specimens including the phantoms were wrapped in parafilm and scanned wet. Mineral density for all tissues were evaluated using the aforementioned procedure.

### 2.6. X-ray fluorescence microscopy of normal and diseased specimens

Normal and diseased alveolar bone, cementum, and dentin specimens were ultrasectioned into blocks and analyzed[9,19]. A 1–2 μm thick section of diseased dentin was used for XRF microscopy to enhance detection of lower Z elements, namely phosphorus, to increase the accuracy of Ca/P measurements. Data was collected using a synchrotron beam at the Stanford Synchrotron Radiation Lightsource (SSRL) on beamline 2–3. Data acquisition was performed using constant parameters with an incident energy beam of 10 keV, dwell time of 25–50 ms per pixel, and a step size of 1 μm. The elemental concentrations were determined by calibration against a set of National Institute of Standards and Testing (NIST) traceable, thin film elemental x-ray fluorescence standards provided by Micromatter (Vancouver, Canada). Corrections to the area-based concentrations (mmol/cm^2^) were performed based on the absorption and attenuation of both the incident (excitatory) x-ray photons (10 keV) and the various elemental fluorescence photons in the matrix of the material. Following calibration, quantitative thickness correction was applied to image maps to account for specimen thickness and generate volume based concentrations (mmol/cm^3^). The input parameters to the thickness correction function (SMAK software)[51] are specimen thickness, specimen absorption length, and fluorescence line energy. Specimen absorption length (based on incident x-ray energy and the specimen's chemical formula, density and thickness) was calculated with X-ray Utils[51–53], and fluorescence line energy (with corresponding yields) was determined from the X-ray Data Booklet[54]. Subsequently, XRF segmentation procedure analogous to micro-CT segmentation was applied to elemental (Ca, P, and Zn) concentration maps. ROIs were directly drawn on the 2D elemental association maps. Areas representing a strong association between Ca and P, and Ca and Zn were marked on the respective tissues by masking clusters in the association maps. Pixels, or concentration values, within each masked region were averaged. The Ca/P and Ca/Zn ratio were calculated by the map math function (SMAK software), which allowed arithmetical operations between elemental maps[51]. A density function was applied to the masked regions to identify local, distinct concentrated zones, indicating homogeneity, or a spread in concentration, indicating heterogeneity between zones. The zones, whether localized or spread, could then be compared with the averaged values within masked ROIs for statistics (see 2.8).

### 2.7. Gravimetric Analysis to determine weights of inorganic matter

Following x-ray attenuation and elemental analyses, ash analysis was performed on four calibration phantoms (0, 250, 500, and 750 mg/cc) and on experimental specimens of normal alveolar bone (N = 3) and normal dentin (N = 3). The ash was weighed and the mineral density in mg/cc was directly calculated from known specimen volumes. Due to the innate irregular geometry of bone, specimens were soaked for at least 3 days in Hank’s solution, and the wet volumes were calculated by scanning at 55 kVp, 8 W, 2X magnification, and at 1400 image projections and post-processing of data by using Amira 5.4.2) (Visage Imaging Inc., San Diego, CA). Nearly perfect geometries of dentin beams and phantoms were calculated after measuring their dimension using a digital Vernier caliper (Mitutoyo ABSOLUTE 500-196-20 Digital Caliper, Stainless Steel, Mitutoyo Corporation, Kawasaki, Japan). Following wet scans, bone and dentin specimens were dried in a vacuum chamber (Bel-art desiccator H42050 and H42051, Bel-Art Products, Wayne, NJ) for 15 days until no relative changes in weight were observed. Experimental specimens were transferred to porcelain crucibles and placed inside a furnace (Ney Neymatic 202 Burnout Oven FS19590, The J.M. Ney Company, Bloomfield, CT) for 48 hrs at 1110 °F whereby all organic material was burnt. The mass of ash was weighed in mg by using a balance (Mettler Toledo Balance Prec 320 G X 0.001 G MS303S) sensitive to 0.001 g (Mettler-Toledo, Inc., Columbus, OH). To note, the size of cementum tissue was small and prevented us from performing an accurate gravimetric analysis for comparison with micro-CT mineral density values.

### 2.8. Statistics

The mean HU value and average mineral density along with standard deviations were calculated for all tissue types and segmented regions within tissues. The micro-CT calibration curve generated from known mineral density phantoms was correlated to ash density of the same phantoms, and the residual (*ê* = *y-ŷ*) was plotted in MATLAB (Matlab 7.10.0, R2010a, The MathWorks, Inc., Natick, MA). A paired t-test was applied on data sets collected before (micro-CT data) and after (gravimetric) ash tests, and on the experimental specimens of normal dentin and adapted bone. Unpaired t-tests were performed to highlight statistical differences across segmented volumes within respective tissues. Note, the Bonferroni correction was applied for dentin data to handle multiple comparisons due to the wide mineral variation present in this tissue. In XRF elemental maps, standard deviations were also calculated for the segmented regions (volumetric concentration values in mmol/cm^3^) in cementum, dentin, and alveolar bone, and a median based ANOVA test was performed between various ROIs within each tissue using the masked statistics function on the MicroAnalysis Toolkit (SMAK software)[51]. Statistical significances were determined (from micro-CT and XRF data) between normal and diseased counterparts of the aforementioned tissue types, and between different tissue types with a 95% confidence interval.

## Results

### 3.1. Mineral density ranges based on segmentation analysis

**Fig 2:**
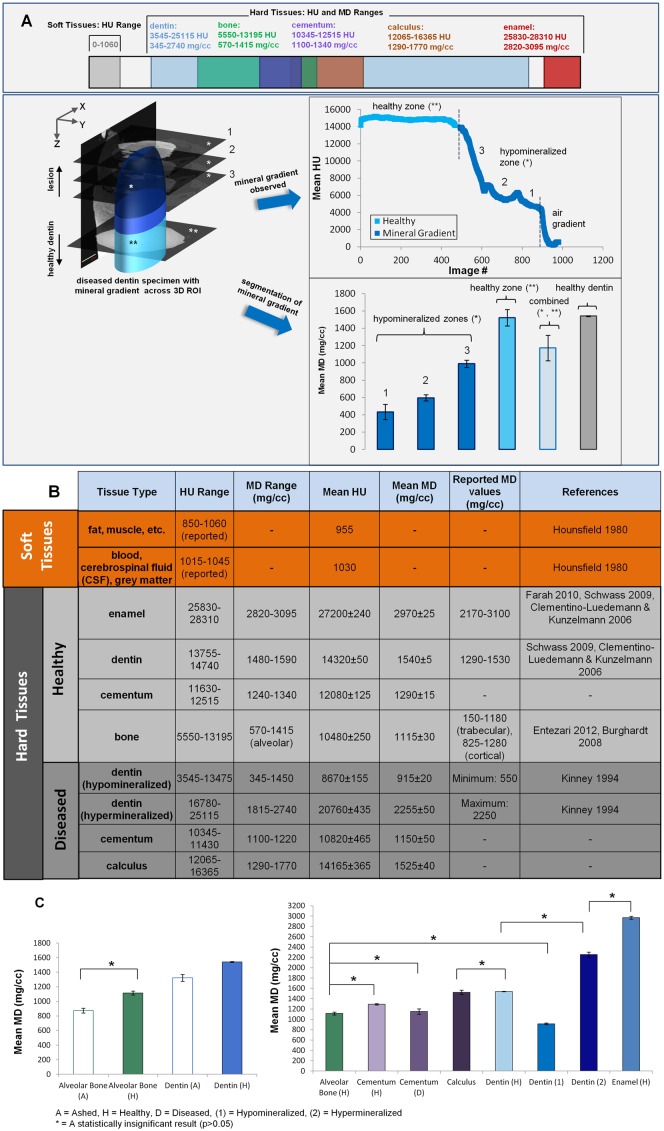
Average HU and MD ranges from experiments and literature. A) UPPER PANEL: Mineral density and HU ranges of hard and soft tissues and regions of overlap amongst tissues are shown. LOWER PANEL: A schematic of segmentation procedure for diseased tissues, where the upper portion of the 3D volume (dark blue, *) represents lesion volume and the lower portion (medium to light blue, **) is a gradient between lesion shifting to a normal healthy tissue. Mineral gradients through the volume of material are identified by computing mineral density from each 2D virtual slice (e.g. 1, 2, and 3 following procedures highlighted in Fig 1B and 1C). Statistically distinct (p<0.05) hypomineralized zones(*) are decoupled from the observed mineral gradient (upper line plot) for the entire diseased region using segmentation (following procedure highlighted in Fig 1D). The bar chart distinguishes each segmented hypomineralized zone against the healthy zone (**), the combined value (average of both healthy and hypomineralized zones) and healthy dentin; B) Summary of mineral density and HU ranges with average values across hard and soft tissues, including healthy and diseased conditions; C) LEFT PANEL: Mineral density of healthy alveolar bone and dentin calculated by gravimetric and micro-CT. RIGHT PANEL: Calculated mineral density of healthy and diseased hard tissues using a micro-computed tomography unit. To attain mineral density from the data, an ROI through samples was taken for healthy tissues (similar to figs B and C), segmentation was applied for diseased tissues (fig D). Segmented data was compared with ashed mineral density. A = ashed, H = Healthy, D = Diseased, (1) = Hypomineralized, (2) = Hypermineralized, * = A statistically insignificant result (p>0.05), while all other groups not indicated with an asterisk are statistically different.

The aforementioned calibration curve using phantoms indicates a linear relationship between mineral density and Hounsfield units (HU): MD(mgHA/cc) = 0.11*(HU) ─ 48.6 (Fig 1A). Ash mineral density of the same phantoms plotted with the micro-CT data gave the relationship, MD(mgHA/cc) = 0.11*(HU) ─ 17.3 indicating linearity and a 0.99 correlation with the original calibration curve shown by the residual (red dotted curve, Fig 1A). The comprehensive ranges of HU and mineral density for all experimental specimens, including normal, diseased, and gravimetric specimens, and literature ranges, are shown in Fig 2. In attaining these ranges, an overview of the segmentation analysis is illustrated in Fig 2A (lower panel) using diseased dentin as an example. The illustration highlights the precision of the segmentation method in its ability to delineate statistically distinct mineral zones (e.g. normal and hypomineralized zones shown), having started with a qualitative guess based on HU value. The mineral density range for normal enamel, dentin, cementum, and bone were: 2820–3095, 1480–1590, 1240–1340, and 570–1415 mg/cc, respectively. Within diseased dentin, the mineral density range for hypomineralized dentin was 345–1450 mg/cc, and the range for hypermineralized dentin was 1815–2740 mg/cc. Diseased cementum and dental calculus exhibited a mineral density range of 1100–1220 mg/cc and 1290–1770 mg/cc, respectively. The mineral density values, compiled in Fig 2B, are also plotted in Fig 2C to illustrate statistical relevance. All groups marked by an asterisk in Fig 2C are statistically similar, while unmarked groups are all statistically distinct.

### 3.2. Mineral gradients and volumes in alveolar bone

**Fig 3:**
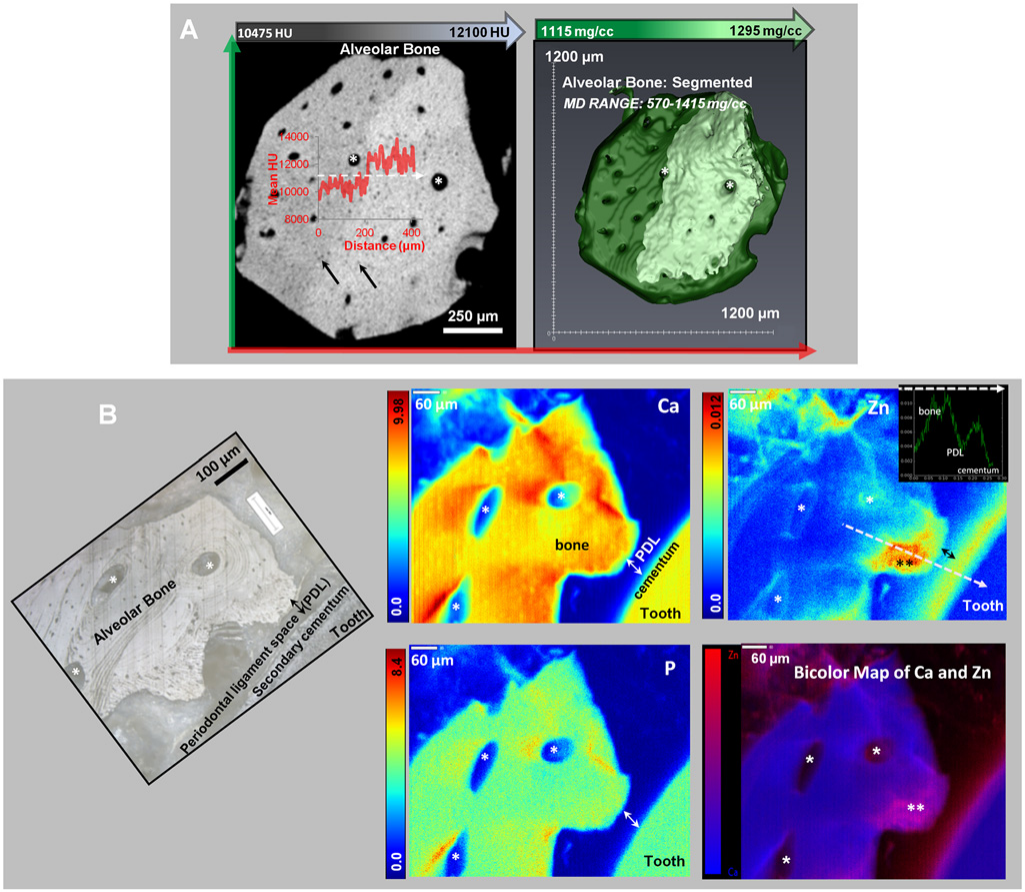
Segmented regions of different mineral densities in alveolar bone. TOP PANEL: A) Left image shows X-ray virtual slice of alveolar bone with higher and lower attenuating regions. Inset illustrates a gradient (red curve) in X-ray attenuation along a line profile (white dotted line). Black arrows represent osteocyte-lacunae spaces, white asterisks are blood vessels or endosteal spaces. Right panel shows 3D segmentation of alveolar bone (MD Range: 1115–1295 mg/cc) with two regions of different mineral densities that complement regions in x-ray attenuation map in bottom panel; BOTTOM PANEL: B) Left image shows a light microscope micrograph at 10X of an ultrasectioned alveolar bone block specimen. White asterisks likely indicate endosteal spaces. Right panels are XRF area maps with concentration gradients in Ca, Zn, and P in mmol/cm^3^. Note that the white asterisks in XRF micrographs indicate the same anatomical locations as in the light microscope image. A representative line profile in the Zn image map (white dotted line, in mmol/cm^3^) vs. distance (mm) from bone to cementum is shown. The black double-asterisk on Zn XRF map indicates a bony protrusion in the PDL-space. The bicolor map of Ca and Zn in the bottom right panel is an overlay of Ca and Zn XRF signals. Within this map, regions dominated by Ca are blue, and regions dominated by Zn are red. An overlap of the two elements is indicated by shades of pink to purple.

micro-CT and XRF imaging within alveolar bone illustrated varying mineral patterns with the presence of gradual and sharp mineral gradients (Fig 3). Two mineralized regions within normal alveolar bone (Fig 3A), as indicated by the line profile, were segmented. A corresponding light microscope image is provided in Fig 3B. Segmented volumes of alveolar bone contained mineral density ranges from 570–1415 (1115±30) mg/cc (N = 4, right image in Fig 3A).

The distribution of Ca, P, and Zn in XRF maps of bone and at the bone-ligament and cementum-ligament interfaces illustrated considerable heterogeneity compared to micro-CT data, in which the mineral content was higher near the bone-PDL interface and near endosteal spaces. In XRF imaging, similar patterns in elemental composition were observed near endosteal and vascular spaces in bone (Fig 3, void spaces with a white asterisk). Zn was predominant at the bone-PDL interface, and segmentation of the interface identified a region of new or adapted bone. The Ca/P ratio within alveolar bone and through the highly concentrated bone-PDL interface (Fig 3B, black asterisks) were both found to be 1.68±0.2, however the corresponding Ca/Zn ratio was 2765±905 and 855±125, respectively. Higher concentrations of Ca and P did not correlate with Zn concentrated regions (Fig 3B). High concentrations of Zn occurred within bone (away from the interface) and were not at vessel locations (Fig 3B). Lacunae (black arrows) in the bone were regions of lower Ca content (Fig 3A).

### 3.3. Mineral gradients in cementum

**Fig 4:**
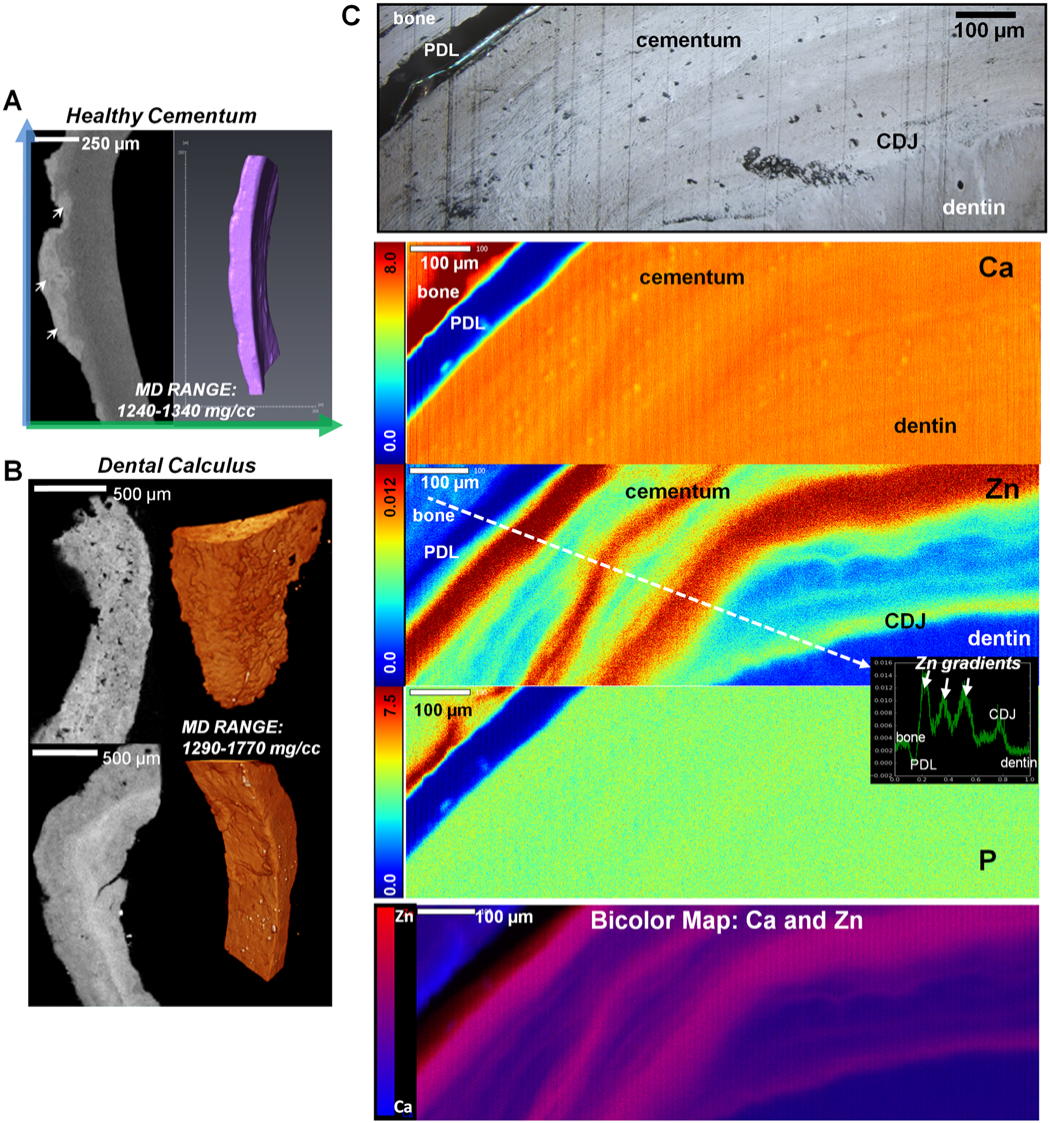
Segmented regions of different mineral densities in cementum. A) X-ray virtual sections of cementum from a periodontically infected tooth illustrates affected cementum with dental calculus (white arrows). Following segmentation (right panel), a narrow mineral density range was identified; B) Mineral gradients in dental calculus with micro-CT 2D virtual sections and 3D volume rendering of supragingival dental calculus (top panel) and subgingival dental calculus (bottom panel). Note that supragingival is porous compared to consistent stratification observed in subgingival while both contain similar ranges of mineral density values (MD Range: 1290–1770 mg/cc); C) Light microscope image at 10X showing various healthy tissues i.e. bone, PDL, cementum, CDJ, and dentin. XRF area maps illustrate concentration gradients in Ca, Zn, and P in mmol/cm^3^. Bottom panel is a bicolor map of Ca and Zn, with blue regions of Ca dominance and red regions of Zn dominance. Note: Same landmarks are shown in all panels. A line profile (white dotted line) in the Zn image map shows Zn concentration (mmol/cm^3^) vs. distance (mm). Line was drawn from bone to dentin, perpendicular to the Zn bands in cementum.

For the volume of specimens analyzed, micro-CT imaging of cementum revealed only a few macro-level differences in mineral density in normal and diseased specimens, however elemental composition differences were detected by XRF, shown in Fig 4. The mineral density range was 1240–1340 (1290±15) mg/cc for normal cementum specimens (N = 5, Fig 4A and 4C) and 1100–1220 (1150±50) mg/cc for disease-affected cementum (N = 5). Affected cementum with calculus (Fig 4B) demonstrated a heterogeneous mineral distribution and cementum resorption. Subgingival calculus (Fig 4B, lower panel) mostly adjacent to cementum contained stratified mineralized matrix compared to a porous heterogeneous configuration exhibited by supragingival calculus (Fig 4B, upper panel). Interestingly, although compositionally and structurally different, the mineral density of calculus overlapped with that of normal dentin with a range of 1290–1770 (1525±40) mg/cc (N = 4).

XRF imaging of cementum (Fig 4C) illustrated Ca, Zn and P bands. The most notable differences, such as the Zn bands, are shown in the mineral gradient profile (white dotted line over the Zn map), and the light micrograph is anatomically the same region as that scanned. The dominant areas of Ca (blue to purple regions) and Zn (red or pink regions) are highlighted in the bicolor map of Ca to Zn. Although Ca and P showed no dominant bands within cementum, both were more evenly distributed throughout cementum than Zn. The Ca/P and Ca/Zn ratios within normal cementum regardless of the presence of dominant features were 1.51±0.22 and 990±430, respectively. Upon segmentation (or masking, see section 2.6) of alternating large Zn bands in cementum, a Ca/Zn ratio of 595±50 was observed for acellular extrinsic layers and 1155±165 for cellular intrinsic layers. The observed lack of Ca and P dominant bands and their even distribution within cementum is shown by the Ca/P ratio of Zn rich and Zn poor layers, or 1.51±0.13 and 1.52±0.13, respectively. In addition, several lacunae spaces could also be identified in the Ca map.

### 3.4. Mineral gradients in dentin

**Fig 5:**
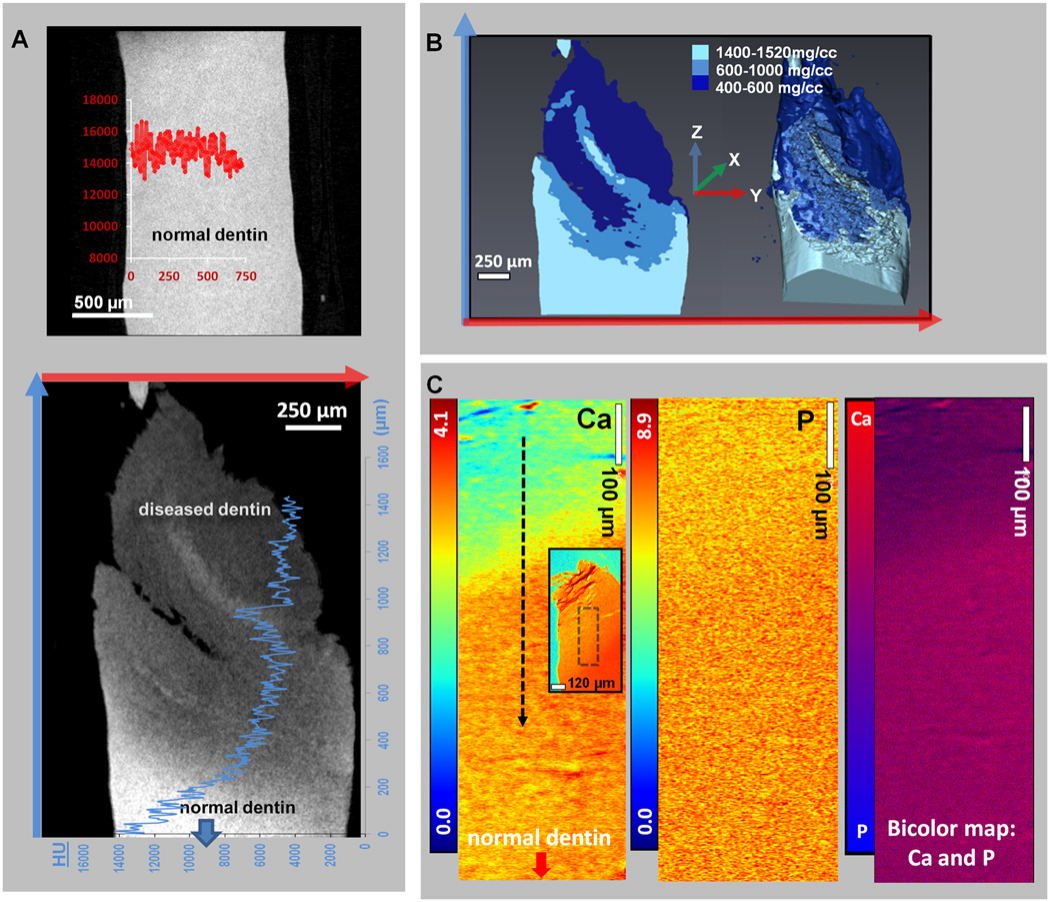
Segmented regions of different mineral densities in dentin. A) TOP PANEL: Virtual section of healthy dentin illustrates a constant HU line profile. LOWER PANEL: Mapped on the micro-CT virtual slice of diseased dentin is the HU gradient along a distance of 1400 μm; B) Segmented volumes within diseased dentin are represented in 2D and 3D. The mineral density range within normal dentin is 1400–1520 mg/cc. Segmented mineral density volumes illustrated two distinct zones of lower mineral content dentin: 400–600 mg/cc and 600–1000 mg/cc; C) Ca and P XRF area maps show concentration gradients (mmol/cm^3^) within diseased dentin regions in a 5 μm thick specimen. Ca and P maps show the region of interest indicated by the black dotted box on the inset, which shows a Ca map of the entire specimen. The bicolor map of Ca and P shows Ca deficiency (purple or blue) in the hypomineralized region (top portion at lesion site) and Ca rich regions (pink) gradually away from the lesion site.

Gradients found in dentin (Fig 5) from micro-CT and XRF data revealed both hypomineralized and hypermineralized regions. S1 Fig is a compilation of the mineral heterogeneity observed in all analyzed diseased dentin specimens, and S1 Movie depicts mineral gradients in the specimen from Fig 5. Hypomineralized and hypermineralized zones were isolated through the observation of discontinuous mineral gradients (compare Figs 2A and 5B), which were segmented into the following categories (N = 5, Fig 5B): lower mineral content (400–600 mg/cc), low mineral content (600–1000 mg/cc), normal dentin (1470–1605 mg/cc), and hypermineralized (1815–2740 mg/cc). In addition, dentin regions were categorized according to the following HU ranges to account for spatial variability: 3,545–13,475 HU (hypomineralized), 13,755–14,740 HU (near normal), and >15,000 HU (hypermineralized).

In XRF maps (Fig 5C), elemental contents of Ca, P, and Zn were compared with the grayscale 2D micro-CT virtual slice (Fig 5A). The micro-CT image was shown to correspond markedly with Ca (due to a higher fluorescence yield) and faintly with P (due to the detection limit for this element). Grayscale changes are analogous to changes in mineral density. The Ca/P ratios in diseased zones (black dotted line in Fig 5C) were 0.32±0.05 and 0.46±0.04, respectively, and the Ca/P ratio of normal dentin was 1.49±0.21. The correlation between Ca and P content (bicolor map, Fig 5C) provides a way to conceptually visualize the segmented hypomineralized regions by micro-CT and XRF.

### 3.5. Micro-CT and microprobe XRF correlation

**Fig 6:**
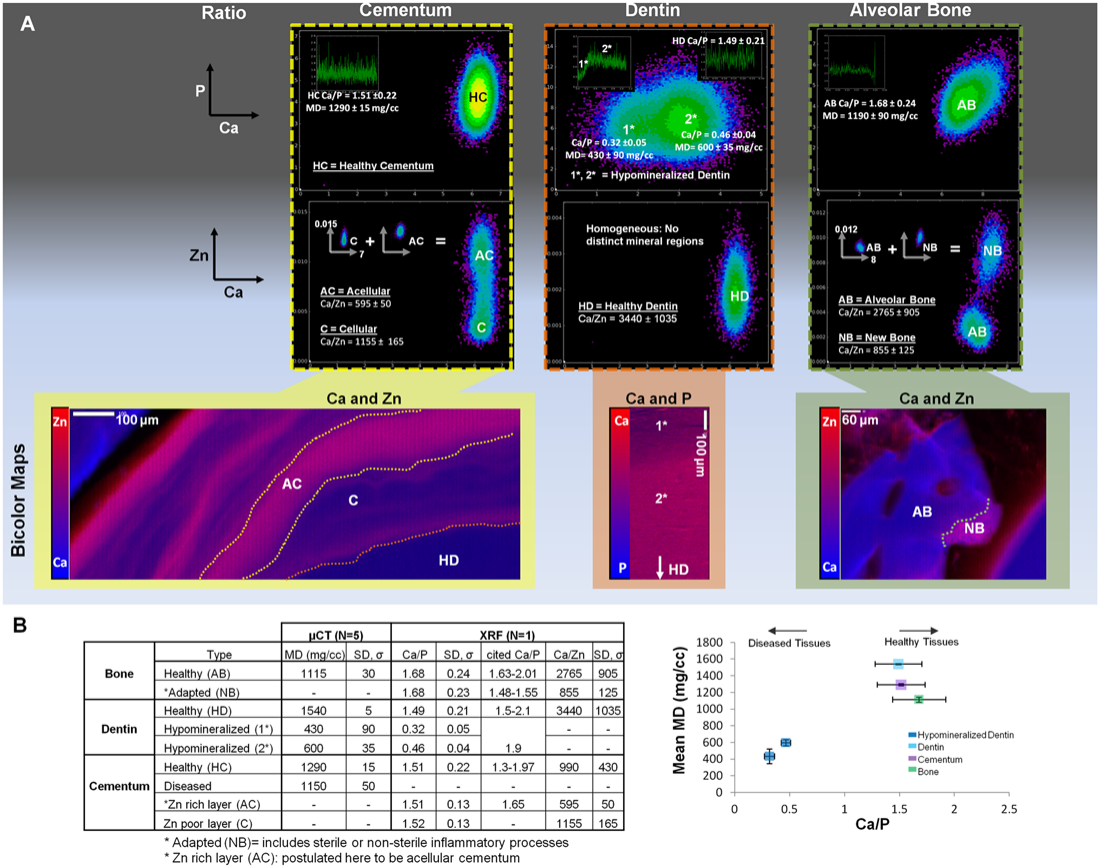
Additional XRF analyses of elemental gradients in cementum, dentin and alveolar bone. A) Correlation (density) plots for cementum, dentin, and alveolar bone show the degree of equivalency between Ca to P and Ca to Zn patterns. Ca/P line plots (insets on the correlation plots) reveal Ca/P ratio range within each tissue. Correlation plots indicate a spread of Ca and P x-ray fluorescence signals in cementum and bone, while distinct zones in hypomineralized dentin (1* and 2*) were found. The reverse was identified for Ca to Zn correlation plots, which show distinct zones in cementum (area maps indicating AC and C) and alveolar bone (area maps indicating AB and NB) due to a range in Zn concentration for a narrow variation in Ca. For Ca/Zn plots, insets indicate summated regions that contributed to the overall spread in Zn vs. Ca signals. XRF bicolor maps (Ca and Zn for cementum and bone, Ca and P for dentin; bottom panel in A) indicate segmented zones from which the correlation plots are derived (C = cellular, AC = acellular, HD = healthy dentin, AB = alveolar bone, NB = new bone). Regions 1* and 2* are both hypomineralized zones, 1* being at the lesion site with severe demineralization (Ca/P = 0.32 ± 0.05) and 2* being directly adjacent to the lesion site with slightly higher mineralization (Ca/P = 0.46 ± 0.04); B) Correlation between micro-CT (N = 5) and XRF data (N = 1) for bone, dentin, and cementum with a corresponding line plot of mean MD vs. Ca/P ratio are shown. No correlation was found between mineral density and the Ca/Zn ratio. Zn dominant regions identified using XRF were not identified using micro-CT For example, an adapted or new bone (NB), Zn rich (AC) and Zn poor (C) layers within cementum were not discerned using micro-CT. The bottom right plot illustrates spread between mineral density and Ca/P ratio and an apparent linearity within diseased and healthy dentin.

The correlation between micro-CT and XRF results is highlighted in Fig 6. Correlation plots for cementum, dentin and alveolar bone (Fig 6A) show localizations corresponding with different mineral zones highlighted in bicolor plots (Ca and Zn or Ca and P). The entirety of Fig 6A outlines the XRF segmentation procedure, and Fig 6B shows the corresponding mineral density values with Ca/P and Ca/Zn ratios for each tissue type. Segmentation analysis using polychromatic radiation (micro-CT) at 10X did not resolve adapted bone nor the Zn-rich and Zn-poor layers in cementum, however these were detected by XRF segmentation analysis. No data was collected for diseased cementum by XRF, and no Ca/Zn ratio for hypomineralized dentin zones could be detected. Since mineral density (mg/cc) and the Ca/P ratio are known to be proportional, the mean MD (mg/cc) vs. Ca/P was plotted for all normal tissues and for hypomineralized dentin to identify any possible correlations.

### 3.6. Statistics

Statistical analyses were conducted both between and within normal and diseased tissues, as illustrated in the plots in Fig 2C. The analyses are based on statistically distinct segmented mineral volumes within individual specimens from micro-CT and XRF data (see section 2.8). Across all normal tissue groups, statistical differences were found except between alveolar bone and cementum (p = 0.19). Between normal and diseased groups, statistical differences (p<0.05) were found in all, except between normal alveolar bone and periodontitis-affected cementum, normal alveolar bone and hypomineralized dentin, healthy dentin and calculus, and healthy enamel and hypermineralized dentin. Gravimetric mineral density data of normal dentin specimens was significantly different (p<0.05) from micro-CT data, however no statistical difference (p = 0.07) was calculated between gravimetric and micro-CT results of normal alveolar bone. Within cementum, a statistical difference in mineral density was observed between normal and diseased states (p<0.01), between normal cementum and calculus (p = 0.03), and between diseased cementum and calculus. Within dentin, statistically distinct segmented volumes were found across hypomineralized, hypermineralized, and normal dentin groups. However, a statistical difference was not strongly apparent between hypermineralized and normal dentin (p = 0.05), and no difference was found between hypermineralized dentin and sound enamel (p>0.05).

## Discussion

An extended perspective offered in this study is that heterogeneity as a result of adaptation (early and later stage events) can be better understood by mapping gradients of mineral concentration in tissue volumes and correlating them with elemental ratios from the same regions. The higher resolving capacity of a micro-CT system despite its polychromatic nature was exploited to gain insights on earlier versus later stages of adaptive biomineralization events within normal and diseased tissues. Specifically for adaptations within diseased tissues, mineral density gradients indicated hypo- and hypermineralized volumes relative to mineral densities of healthy tissues. As a result, this study provides insights on adaptive biomineralization processes that prompted the measured densities and elemental distributions. However, prior to discussing the MD and elemental results related to normal and diseased tissues, it is important to discuss experimental limitations despite the use of state-of-the-art technology in this study.

Experimental limitations included varying specimen thickness and its potential effects on spatial mapping of 1) mineral density related attenuation for normal and adapted volumes; 2) Ca, P, and Zn elemental distribution through x-ray fluorescence signals; 3) hypo- and hypermineralized volumes within adapted dentin where significant differences in Ca/P ratios were observed[55,56] including higher concentrations of elemental Zn. In addition, 4) spatial sensitivity of correlative results related to spatial resolving capacity of polychromatic and monochromatic radiations will be discussed.

Specimen thickness can affect both x-ray attenuation and fluorescence signals. Spatial resolution of these signals is limited by respective instrument sensitivities. Monochromatic radiation[57,58] from a synchrotron facility can be tuned to above and below absorption edges of an element of interest and its spatial resolution within the matrix[59,60] can be determined. However, benchtop micro-CT systems are polychromatic and as a result the attenuation is a summation of absorption of x-rays of varying energies by elements representative of organic and inorganic components within voxels of tomograms. Since the energy of the x-rays is minimally tunable in a bench top unit, the attenuation between one voxel to the next could also change due to density of elements packed in a non-uniformly thick biological tissue. Attenuation values reported to date assume uniform specimen thickness, but it is a challenge with biological specimens of varying mineral densities, i.e. an innate heterogeneity exists due to various macromolecules and elements present in a tissue. Hence, the thickness of a tissue although reported uniform are indeed non-uniform and sectioning by an ultra-microtome or by focusing an ion beam will generate a specimen with non-uniform thickness. Given such a scenario, x-ray attenuation is not necessarily due to mineral exclusively, but is due to the effect of mineral within an organic matrix of an uncertain thickness, all of which can contribute to its attenuation. To investigate the error due to thickness, we used a conical specimen of pure hydroxyapatite (Fig 1C, 'HA cone' curve) vs. a cylindrical pure hydroxyapatite specimen (Fig 1C, 'phantom pure HA' curve). Attenuation differences revealed a variance less than 10% using polychromatic benchtop micro-CT. In other words, the conical specimen with its extreme thickness variation was scanned to identify the worst possible systematic effects that could arise from the calibration procedure. Since calibration phantoms match the field of view coverage of experimental specimens, the curvature in the HA cone profile represents the worst possible variation in the calibration, and was found to be acceptable (<10%). However, the enamel specimen is the hardest material and need not have the same characteristics of a relatively softer yet mineralized tissue where “relief in tissues” can be created upon sectioning of thin specimens from blocks, and that the tissue relief in and of itself can cause density variations.

Similar arguments related to specimen dimension hold true for element mapping by recording x-ray fluorescence through the use of microprobe XRF technique but using monochromatic X-rays. Given that the specimens used were in the form of blocks, and the mean free path length of Ca and P is less than the specimen thickness, it is likely that some fluorescence signal is absorbed before it is received by the detector. Hence, thinner specimens are recommended for Ca and P (specifically if they exist in lower quantities), when compared to Zn whose mean free path length is significantly greater than Ca and P. We attempted to map the distribution of Ca, P, and Zn from blocks and thinner sections obtained from the blocks. While specimens were of varying thickness values and physical densities, they were of an adequate size to provide a representative pattern of elemental composition and/or a mineral density. In addition, the varying thickness was accounted for using an algorithm in XRF analysis as noted in section 2.5. As a result, the correlation between XRF and micro-CT techniques was exploited through segmentation processes. Ca and P are the main elements in hydroxyapatite contained in enamel, cementum, dentin, and bone. In addition, we identified Zn, and the reason for its existence in adapted tissue segments to date is limited to a postulate[19,59,61,62] which should be investigated. Based on the bicolor maps in Fig 6A, interestingly, the foci of Ca and Zn in cementum and bone exist as different patterns, questioning the possibility of different regulators of mineralization during adaptation of respective tissues.

As stated earlier, the regulation of mineral formation can be in two separate forms, or a combination of the two; biologically controlled mineralization occurring due to natural physiologic and/or endogenous events, or biologically induced mineralization due to exogenous or pathological stimuli[12]. Through analytical techniques we mapped events representative of a single time point that resulted due to a long cascade of orchestrated events within organisms. Added to the inherent complexity is the need to also understand the link between the two fundamental forms of mineralization through extension of analytical techniques that include crystallography, x-ray diffraction, texture, followed by identifying chemical environments for elements using x-ray absorption near edge spectroscopy, and Raman spectroscopy. These techniques when complemented and correlated with biochemical assays specific to tissue turnover rates could elucidate the mechanistic processes of adaptive biomineralization involving Ca, P and Zn ions, and potentially other elements such a commonly identified Mg, K, Cu, Sr, or Pb[30–32,38,40,44]. It should be noted that within the broad classification of biomineralization processes could also lay the elusive switch to biologically induced from being biologically controlled. It is plausible that the switch can prompt secondary formations within tissues and likely can be turned on due to external stimuli prompting adaptive biomineralization process. Examples of adaptive biomineralization (Fig 6) include the observed modeled bone (Ca/Zn = 855), incremental layers of cementum (Ca/Zn = 595) indicated by higher concentrated regions of Zn, and Ca/P ratios of hypomineralized dentin volumes (0.32–0.46). Significantly lower Ca/Zn ratios within adapted tissue regions compared with their normal counterparts suggests not only the importance of Zn for biologically controlled events but also indicates Zn may play a significant role at the onset and progression of pathologic biomineralization.

In this study, detectable amounts of zinc were observed in tissues with secondary formations, including bundle bone growing from lamellar bone, or osteonal bone growing from the primary lamellar bone[19,63]. Secondary events are marked distinctly by significant variations in structure and mineral density rendering either a lower or a higher attenuation relative to primary bone (Fig 3A). A point to note is that, the gravimetric analysis of alveolar bone is consistent with the range of mineral density provided in literature (Fig 2B); 150–1280 mg/cc (trabecular and cortical)[18,24]. Because mineral can become crystalline following an amorphous phase, mineral density between primary and secondary formations can be heterogeneous (Fig 3A and 3B). However, it is uncertain to what extent the higher concentration surrounding the void spaces in all XRF maps (white asterisks in Fig 3B) is related to physiological effects or simply due to signals picked up from subsurface layers. In the Ca and Zn bicolor map for alveolar bone (Fig 3B), the double asterisks may indicate a region of higher tissue turnover (an "adapted bone" region or an abnormality in biomechanical function), indicated by higher Zn content. The Ca/P ratio was 1.68 ± 0.2 in normal and adapted bone, which is near the literature Ca/P ratio of 1.63–2.01 for alveolar bone detected using an energy-dispersive X-ray (EDX) micro-analysis and transmission electron microscopy (TEM)[31,32] techniques. However, it should be noted, that the larger sampling volume by XRF (a few mm^3^) using synchrotron monochromatic radiation could reflect the Ca/P ratio accurately compared to the results from an electron probe in EDX technique due its inherent limitations

Similarly, within secondary cementum, mineralized regions are influenced by the presence of incremental growth lines and are hypothesized to exist due to a significant shift in metabolic activities that coincide with the presence or absence of cementocytes-lacunae. The spatial arrangement and mineral content of incremental lines as a lifelong apposition was found to vary along the root[4,64,65]. Micro-CT results at 10X magnification did not illustrate stratified cementum; however faint mineralized streaks within cementum (Fig 4A) were observed. It could be that the specimen volume is minimal as higher resolution micro-CT scans of cementum in the past revealed distinct lamellae within cementum[66]. XRF imaging complements micro-CT data by demarcating specific element-dominating regions. Light microscopy indicated normal cementum at the apical portion of the root with discrete cellular and acellular demarcations which were complemented by non-discrete overlap of Ca, P, and Zn bands (bicolor map, Fig 4C and Fig 6A) as detected by microprobe XRF technique. From the data, we postulate that the hypermineralized regions (Fig 4C, bright regions in the light microscope image, dark red/orange zones in the Ca and Zn maps, yellow zones in the P map, red and pink zones in the bicolor map) may represent a dominance of acellular extrinsic fiber cementum, whereas the hypomineralized regions in-between (Fig 4C, blue or purple regions in the cementum bicolor map) may correspond to cellular intrinsic fiber cementum[7]. As described earlier, the functional attachment of the root to bone is not limited to coronal cementum[67], and that attachment is also carried over to secondary cementum. In addition, the Ca/P ratio within cementum showed a relatively constant range (1.51±0.22), with no significant difference between layers of lower and higher mineral contents. The experimental values were well within the reported range of 1.3–1.97 and closer to 1.65, an ionic ratio of acellular cementum detected by EDX micro-analysis[30,31].

Although not apparent qualitatively, quantitative results from micro-CT illustrated distinct mineral density ranges for periodontitis-affected cementum (1100–1220 mg/cc) and dental calculus (1290–1770 mg/cc) in Fig 4B. The mineral density of normal cementum (1240–1340 mg/cc) was also identified. In addition, higher Ca content of the surface layer of cementum was observed by XRF, comparable to the densely mineralized surface layer observed by our previous studies[8]. Cementum is often affected by concretion due to cascade of biochemical and physicochemical effects as a result of yet another acerbated stimulus, such as periodontitis. As reported previously[67], we postulate that periodontitis can prompt bioapatite formation as a concerted effort between biologically induced and controlled processes[8]. As such additional concretion of cementum layers, specifically those closer to the PDL can occur in addition to subgingival calculus formation which could be induced by bacteria. While secondary events could be the potential cause for observed mineral density ranges in cementum, there underlies a different set of secondary events that can potentiate hypomineralized and hypermineralized zones within dentin.

Mineral gradient patterns were also identified by others in primary, secondary, and tertiary forms of dentin[68,69]. Given that the specimens are from 40–60 year old male individuals, the aforementioned types of dentin could exist in the following singular or mixed forms: 1) hypomineralized dentin due to carious or non-carious lesions (i.e. trauma, mechanical stimuli) or 2) hypermineralized dentin in the form of sclerosis, tertiary dentin (reactive or reparative), or with precipitates of apatite[7,56,68,69] observed as bands, streaks, and precipitates relative to less attenuating diseased dentin (Fig 5A, S1 Fig). Our results could represent all forms of dentin, and the degree of heterogeneity illustrated within is a function of the magnification at which the specimens were scanned using the X-ray microscope. To note, the reported mineral density values are for hydrated volumes of tissues. Furthermore, what is largely unknown is if secondary non-carious dentin becomes hypomineralized or hypermineralized when compared to primary dentin. Mineralization effects due to aging in non-carious dentin have not been thoroughly understood, however an increase in the amount of mineral in peritubular dentin with age, and thus in the dentin tissue, has been identified[70–72] with a possible shift in the normal to a hypermineralized range.

The Ca/P ratio along with correlation plots, i.e. density functions (Fig 6A) indicating areas of concentrated levels of Ca and P were already noted in section 3.4. The relatively uniform distribution of P concentration and decreased Ca concentration through a diseased region could possibly be due to low fluorescence yield as a result of lighter and heavier elements. The Ca/P ratio literature range of normal dentin is between 1.5–2.1[33,73], and 1.9 for hypomineralized dentin[33]. The significant difference in our Ca/P ratio of diseased dentin (0.32±0.05 to 0.46±0.04) could indicate the severity of the disease, while the Ca/P ratio we reported for normal dentin (1.49±0.21) matched the literature range[33,73]. The significant change in Ca/P ratio is thought to be altered rates of dissolution due to carious acids accompanied by varying degrees of tubular closure, and formation of loose structures[74]. The corroborating evidence for diffusion gradients to prompt dissolution is the observed lowest mineralized regions surrounding the lesion site (345–600 mg/cc), with the advancing fronts of dissolution following tubule orientation[75]. Other characteristics possibly adding to the heterogeneity of diseased dentin include the observed band region (930–1230 mg/cc) within globules of higher mineral density (higher attenuating regions equivalent to interglobular dentin) and increased tubule occlusion[76]. The segmented regions in 2D and 3D are shown in S2 Movie and S3 Movie.

Hypermineralization in carious dentin can occur due to sclerosis known to result from persistent bacterial, chemical attack, and/or aberrant mechanical stimuli, all of which can prompt odontoblastic cells to occlude tubules as a defense response to protect the dental pulp[77]. For example, significantly different hypermineralized bands or streaks (2255±50 mg/cc) relative to surrounding hypomineralized regions (915±20 mg/cc) within active lesions (Fig 5, S1A–S1C Fig) are another form of sclerosis (S1B Fig), and were observed by others using a light[69] and transmission electron microscope[78] and are in close agreement with our micro-CT results. Given the degree of variation in mineral density in sclerotic dentin (hypermineralized subregions of 1815 and 2740 mg/cc within a normal dentin region of 1480–1590 mg/cc (Fig 2B, S1B and S1D Fig), it is conceivable that the observed darker regions of dentin (from a light microscope), and hypermineralized as per the micro-CT could be a type of reactive sclerotic dentin caused by an influx of calcium and phosphate ions in the oral environment[69]. It is plausible that the heterogeneous nature of specimens and micro-CT limitations can result in the observed variance between gravimetric and micro-CT. The variance may also be due to the contribution of intensity variations related to organic matter during micro-CT calibration. Despite the statistical differences, confidence was gained in the evaluated mineral density ranges as they agreed with the overlap ranges in the literature (Fig 2B); 1290–1530 mg/cc reported for normal dentin[16,23], 2170–3100 mg/cc for normal enamel[16,79], a minimum of 550 mg/cc for hypomineralized dentin and up to 2250 mg/cc for hypermineralized dentin[26]. Other forms of hypermineralized regions in carious dentin can be tertiary dentin or apatite precipitation (2200–2750 mg/cc, S1A–S1C Fig).

Zn in amounts of one-hundredth to one-thousandth in concentration to that of Ca and P was observed in bone and cementum. The unique patterns of Zn (Figs 3–6) that can be partially correlated with Ca and P, could be caused by activation of matrix metalloproteinases (MMPs)[80–82]. In studies by others, heavy metal exposure (Zn, Cd, Pb, Cu) has been shown to reflect an environmental or dietary response in teeth including in humans[83,84]. In addition, it was postulated by Martin et al.[38] that higher levels of trace metals such as Zn in teeth indicate a pathologic state, and that MMP activity during inflammation can exacerbate the effects of disease, e.g. periodontal disease[85], caries[86], cancer[87], or cardiovascular disease[88] by destroying collagen and other proteins that form the extracellular matrix[89]. The lack of Zn in diseased compared to normal regions of dentin could possibly be due to a significantly lower sampling volume of the specimen, which had a thickness of ~5 μm compared with block specimens of cementum with thickness ~3mm. Zn bands correlated with acellular cementum layers, and the Ca/Zn ratio plot (Fig 4C and Fig 6A) indicating the alternating MMP activity during tissue turnover and remodeling and commonly described as incremental layers[7]. In the absence of 3D elemental mapping, a correlation between micro-CT and XRF data through a quantitative basis enabled a proof-of-concept to be established (Fig 6). The complementary data provides insights on pathological states in tissues.

## Conclusions

Results from this study provide an extended perspective on mineral density gradients by correlating with concentrations of mineral forming elements. Additionally, the elemental foot print is also essential information that can aid in elucidating mechanistic processes responsible for pathologic formations. Using high resolution x-ray microscopy followed by segmentation of higher or lower mineral zones, it is possible to discretize mineral gradients in 3D and correlate them with elemental composition albeit lack of voxel to voxel spatial sensitivity due to the nature of poly- and mono-chromatic radiation used in the two experimental techniques. Higher resolving capacity of instrumentation allows segmentation based on mineral density variation and the information obtained is advantageous compared to the common method of reporting one value indicating a homogenous mineral distribution. Following calibration of both instruments, digital segmentation highlights the complementary importance of both techniques; micro-CT segmentation coupled with Ca/P and Ca/Zn ratios as obtained using microprobe XRF technique to provide insights to normal and pathologic biomineralization processes within respective tissues. Through the analyses, we have also shown that it is possible to correlate mineral density (mg/cc) and the Ca/P ratio, however further studies would be required to examine their known theoretical relation, involving micro X-ray absorption near edge structure (μXANES in 2D and 3D tomography) and Raman spectroscopy. Such techniques can probe Zn species in adapted regions related to Ca content, and the Ca/P ratio to provide a plausible basis for pathologic biomineralization. A systematic study in the future highlighting a possible correlation between mineral density and acerbated stimuli such as disease and/or mechanical loads can provide insights to the role of various elements in building minerals and degree of mineralization necessary for tissues to bear functional loads.

## Supporting Information

S1 Fig Experimental diseased dentin specimens and types of lesions. a) carious dentin with active lesion and a dentin beam sectioned from coronal portion of a tooth has revealed sclerotic dentin and hypermineralized zone; b) sclerotic dentin is highlighted by its unique streaking pattern in agreement with observations made by others (Schüpbach 1992); c) periodontally affected tooth with various mineralized zones; d) a carious dentin beam used for XRF imaging that illustrates a hypomineralized zone in dentin towards the lesion site. XRF images of a different dentin slice (5 μm thick) showing Ca and P area maps along with light microscope images of a block dentin specimen affected with caries.
(TIF)

S1 Movie Micro-CT virtual slices through lesion dentin specimen from Fig 5. (MPG)

S2 Movie Lesion dentin specimen from S1 Fig. with 2D segmentation on hypomineralized and hypermineralized zones. (MOV)

S3 Movie Lesion dentin specimen from S1 Fig. with 3D reconstructed volumes of differing mineral densities. (MOV)

S1 Supplemental Information Supplemental Information. (DOCX)

## References

[1] Carter Y, Thomas CDL, Clement JG, Cooper DML. Femoral osteocyte lacunar density, volume and morphology in women across the lifespan. J Struct Biol. 2013;183: 519–526. 10.1016/j.jsb.2013.07.004 23872433

[2] Robling AG, Castillo AB, Turner CH. Biomechanical and molecular regulation of bone remodeling. Annu Rev Biomed Eng. 2006;8: 455–498. 16834564 10.1146/annurev.bioeng.8.061505.095721

[3] Frost HM. Dynamics of bone remodeling. Bone Biodyn. 1964;315: 1–12.

[4] Bosshardt DD, Selvig KA. Dental cementum: the dynamic tissue covering of the root. Periodontol 2000. 1997;13: 41–75. 10.1111/j.1600-0757.1997.tb00095.x 9567923

[5] Lieberman DE. The Biological Basis for Seasonal Increments in Dental Cementum and Their Application to Archaeological Research. J Archaeol Sci. 1994;21: 525–539. 10.1006/jasc.1994.1052

[6] Jin Y, Yip H-K. Supragingival Calculus: Formation and Control. Crit Rev Oral Biol Med. 2002;13: 426–441. 10.1177/154411130201300506 12393761

[7] Ten Cate A. Oral Histology: Development, Structure and Function. 5th ed St. Louis, MO: Mosby Inc.; 1998 pp. 497.

[8] Lin JD, Aloni S, Altoe V, Webb SM, Ryder MI, Ho SP. Elastic discontinuity due to ectopic calcification in a human fibrous joint. Acta Biomater. 2013;9: 4787–4795. 10.1016/j.actbio.2012.08.021 22917805 PMC3529509

[9] Ho SP, Balooch M, Goodis HE, Marshall GW, Marshall SJ. Ultrastructure and nanomechanical properties of cementum dentin junction. J Biomed Mater Res A. 2004; 68: 343–351. 14704976 10.1002/jbm.a.20061

[10] Kinney JH, Habelitz S, Marshall SJ, Marshall GW. The Importance of Intrafibrillar Mineralization of Collagen on the Mechanical Properties of Dentin. J Dent Res. 2003;82: 957–961. 10.1177/154405910308201204 14630894

[11] Hargreaves KM. Seltzer and Bender’s Dental Pulp. Second ed Goodis HE, Tay FR, editors. Hanover Park, IL: Quintessence Pub Co; 2012 pp. 512.

[12] Weiner S, Dove PM. An overview of biomineralization processes and the problem of the vital effect. Rev Mineral Geochem. 2003;54: 1–29.

[13] Boskey AL. Biomineralization: An Overview. Connect Tissue Res. 2003;44: 5–9. 10.1080/03008200390152007 12952166

[14] Swain MV, Xue J. State of the Art of Micro-CT Applications in Dental Research. Int J Oral Sci. 2009;1: 177–188. 10.4248/IJOS09031 20690421 PMC3470105

[15] Davis GR, Wong FSL. X-ray microtomography of bones and teeth. Physiol Meas. 1996;17: 121 10.1088/0967-3334/17/3/001 8870055

[16] Clementino-Luedemann TNR, Kunzelmann K-H. Mineral concentration of natural human teeth by a commercial micro-CT. Dent Mater J. 2006;25: 113–119. 16706305 10.4012/dmj.25.113

[17] Kazakia GJ, Burghardt AJ, Cheung S, Majumdar S. Assessment of bone tissue mineralization by conventional x-ray microcomputed tomography: Comparison with synchrotron radiation microcomputed tomography and ash measurements. Med Phys. 2008;35: 3170–3179. 10.1118/1.2924210 18697542 PMC2673562

[18] Entezari V, Vartanians V, Zurakowski D, Patel N, Fajardo RJ, Müller R, et al Further improvements on the factors affecting bone mineral density measured by quantitative micro-computed tomography. Bone. 2012;50: 611–618. 10.1016/j.bone.2011.10.004 22044640

[19] Hurng JM, Kurylo MP, Marshall GW, Webb SM, Ryder MI, Ho SP. Discontinuities in the human bone–PDL–cementum complex. Biomaterials. 2011;32: 7106–7117. 10.1016/j.biomaterials.2011.06.021 21774982 PMC3185383

[20] Kim I, Paik K-S, Lee S-P. Quantitative evaluation of the accuracy of micro-computed tomography in tooth measurement. Clin Anat. 2007;20: 27–34. 10.1002/ca.20265 16372341

[21] Rossi M, Casali F, Romani D, Bondioli L, Macchiarelli R, Rook L, et al MicroCT Scan in paleobiology: application to the study of dental tissues. Nucl Instrum Meth B. 2004;213: 747–750. 10.1016/S0168-583X(03)01697-5

[22] Neves A de A, Coutinho E, Vivan Cardoso M, Jaecques SV, Van Meerbeek B. Micro-CT based quantitative evaluation of caries excavation. Dent Mater. 2010;26: 579–588. 10.1016/j.dental.2010.01.012 20347481

[23] Schwass DR, Swain MV, Purton DG, Leichter JW. A System of Calibrating Microtomography for Use in Caries Research. Caries Res. 2009;43: 314–321. 10.1159/000226230 19556791

[24] Burghardt AJ, Kazakia GJ, Laib A, Majumdar S. Quantitative Assessment of Bone Tissue Mineralization with Polychromatic Micro-Computed Tomography. Calcif Tissue Int. 2008;83: 129–138. 10.1007/s00223-008-9158-x 18685797 PMC2801565

[25] Dowker SEP, Elliott JC, Davis GR, Wassif HS. Longitudinal study of the three-dimensional development of subsurface enamel lesions during in vitro demineralisation. Caries Res. 2003;37: 237–245. 70865 12771498 10.1159/000070865

[26] Kinney J, Marshall GW Jr, Marshall SJ. Three-dimensional mapping of mineral densities in carious dentin: theory and method. Scanning Microsc. 1993;8: 197–204; discussion 204–205. 7701295

[27] Pugach MK, Strother J, Darling CL, Fried D, Gansky SA, Marshall SJ, et al Dentin Caries Zones: Mineral, Structure, and Properties. J Dent Res. 2009;88: 71–76. 10.1177/0022034508327552 19131321 PMC2759645

[28] Burwell AK, Thula-Mata T, Gower LB, Habeliz S, Kurylo M, Ho SP, et al Functional Remineralization of Dentin Lesions Using Polymer-Induced Liquid-Precursor Process. PLoS ONE. 2012;7: e38852 10.1371/journal.pone.0038852 22719965 PMC3374775

[29] Davis GR, Evershed ANZ, Mills D. Quantitative high contrast X-ray microtomography for dental research. J Dent. 2013;41: 475–482. 10.1016/j.jdent.2013.01.010 23380275

[30] Alvarez-Pérez MA, Alvarez-Fregoso O, Ortiz-López J, Arzate H. X-Ray Microanalysis of Human Cementum. Microsc Microanal. 2005;11: 313–318. 10.1017/S1431927605050221 16079015

[31] Arzate H, Alvarez-Perez MA, Alvarez-Fregoso O, Wusterhaus-Chávez A, Reyes-Gasga J, Ximenez-Fyvie LA. Research Reports Biomaterials & Bioengineering Electron Microscopy, Micro-analysis, and X-ray Diffraction Characterization of the Mineral-like Tissue Deposited by Human Cementum Tumor-derived Cells. J Dent Res. 2000;79: 28–34. 10.1177/00220345000790010301 10690657

[32] Kyriazis V, Tzaphlidou M. Skeletal Calcium/Phosphorus Ratio Measuring Techniques and Results. I. Microscopy and Microtomography. Sci World J. 2004;4: 1027–1034. 10.1100/tsw.2004.200 PMC5956472 15578127

[33] Arnold WH, Konopka S, Gaengler P. Qualitative and Quantitative Assessment of Intratubular Dentin Formation in Human Natural Carious Lesions. Calcif Tissue Int. 2001;69: 268–273. 10.1007/s002230020023 11768196

[34] Hayashizaki J, Ban S, Nakagaki H, Okumura A, Yoshii S, Robinson C. Site specific mineral composition and microstructure of human supra-gingival dental calculus. Arch Oral Biol. 2008;53: 168–174. 10.1016/j.archoralbio.2007.09.003 17964529

[35] Abraham J, Grenón M, Sánchez HJ, Pérez CA, Barrea RA. Spectrochemical Analysis of Dental Calculus by Synchrotron Radiation X-ray Fluorescence. Anal Chem. 2002;74: 324–329. 10.1021/ac0106389 11811404

[36] Abraham J, Grenón M, Sánchez HJ, Pérez C, Barrea R. A case study of elemental and structural composition of dental calculus during several stages of maturation using SRXRF. J Biomed Mater Res A. 2005;75A: 623–628. 10.1002/jbm.a.30484 16116601

[37] Drzazga Z, Michalik K, Maciejewska K, Trzeciak H, Kaszuba M. Role of endogenous zinc in bones of newborn rats. BioFactors. 2007;30: 243–248. 18607073 10.1002/biof.5520300405

[38] Martin RR, Naftel SJ, Nelson AJ, Edwards M, Mithoowani H, Stakiw J. Synchrotron radiation analysis of possible correlations between metal status in human cementum and periodontal disease. J Synchrotron Radiat. 2010;17: 263–267. 10.1107/S0909049509052807 20157281

[39] Aaseth J, Boivin G, Andersen O. Osteoporosis and trace elements—An overview. J Trace Elem Med Biol. 2012;26: 149–152. 10.1016/j.jtemb.2012.03.017 22575536

[40] Oprea C, Szalanski PJ, Gustova MV, Oprea IA, Buzguta V. Multivariate comparison of elemental concentrations in human teeth. Appl Radiat Isot. 2009;67: 2142–2145. 10.1016/j.apradiso.2009.04.017 19497757

[41] Pemmer B, Roschger A, Wastl A, Hofstaetter JG, Wobrauschek P, Simon R, et al Spatial distribution of the trace elements zinc, strontium and lead in human bone tissue. Bone. 2013;57: 184–193. 10.1016/j.bone.2013.07.038 23932972 PMC3807669

[42] Martin RR, Naftel SJ, Nelson AJ, Feilen AB, Narvaez A. Synchrotron X-ray fluorescence and trace metals in the cementum rings of human teeth. J Environ Monit. 2004;6: 783–786. 10.1039/B408525F 15480490

[43] Martin RR, Naftel SJ, Nelson AJ, Feilen AB, Narvaez A. Metal distributions in the cementum rings of human teeth: possible depositional chronologies and diagenesis. J Archaeol Sci. 2007;34: 936–945. 10.1016/j.jas.2006.09.018

[44] Knuuttila M, Lappalainen R, Kontturi-Närhi V. Concentrations of Ca, Mg, Mn, Sr and Zn in supra- and subgingivalcalculus. Eur J Oral Sci. 1979;87: 192–196. 10.1111/j.1600-0722.1979.tb00672.x 293882

[45] Knuuttila M, Lappalainen R, Kontturi-Närhi V. Effect of Zn and Mg on the formation of whitlockite in human subgingival calculus. Eur J Oral Sci. 1980;88: 513–516. 10.1111/j.1600-0722.1980.tb01261.x 6941365

[46] Grøn P, Van Campen GJ. Mineral composition of human dental calculus. Helv Odontol Acta. 1967;11: 71–74. 6022588

[47] Hounsfield GN. Computed Medical Imaging. Science. 1980;210: 22–28. 6997993 10.1126/science.6997993

[48] Ridler TW, Calvard S. Picture thresholding using an iterative selection method. IEEE Trans Syst Man Cybern. 1978;8: 630–632.

[49] Digabel H, Lantuéjoul C. Iterative algorithms. Quantitative Analysis of Microstructures in Material Science, Biology and Medicine Proc. 2nd European Symp. Stuttgart, West Germany: Riederer Verlag; 1978 Vol. 19 pp. 8.

[50] Roerdink JB, Meijster A. The watershed transform: Definitions, algorithms and parallelization strategies. Fundam Informaticae. 2000;41: 187–228.

[51] Webb S. The MicroAnalysis Toolkit: X-ray Fluorescence Image Processing Software AIP Conference Proceedings. AIP Publishing; 2011 Vol. 1365. pp. 196–199.

[52] Elam WT, Ravel BD, Sieber JR. A new atomic database for X-ray spectroscopic calculations. Radiat Phys Chem. 2002;63: 121–128. 10.1016/S0969-806X(01)00227-4

[53] McMaster WH, Del Grande NK, Mallett JH, Hubbell JH. Compilation of X-Ray Cross Sections, Section III. UCRL-50174 Lawrence Radiat Lab, Livermore CA; 1969.

[54] Thompson A, Lindau I, Attwood D, Liu Y, Gullikson E, Pianetta P, et al X-ray data booklet. LBNL/PUB-490 Rev. 3, Lawrence Berkeley National Lab, Berkeley CA; 2009.

[55] Fusayama T. Two layers of carious dentin; diagnosis and treatment. Oper Dent. 1978;4: 63–70. 296808

[56] Magloire H, Romeas A, Melin M, Couble ML, Bleicher F, Farges JC. Molecular Regulation of Odontoblast Activity under Dentin Injury. Adv Dent Res. 2001;15: 46–50. 10.1177/08959374010150011201 12640739

[57] Andrews JC, Brennan S, Liu Y, Pianetta P, Almeida EA, van der Meulen MC, et al Full-field transmission x-ray microscopy for bio-imaging. J Phys Conf Ser. 2009;186: 012081 10.1088/1742-6596/186/1/012081 PMC2811963 20111669

[58] Andrews JC, Meirer F, Liu Y, Mester Z, Pianetta P. Transmission X-ray microscopy for full-field nano imaging of biomaterials. Microsc Res Tech. 2011;74: 671–681. 10.1002/jemt.20907 20734414 PMC2992572

[59] Ho SP, Kurylo MP, Grandfield K, Hurng J, Herber R-P, Ryder MI, et al The plastic nature of the human bone–periodontal ligament–tooth fibrous joint. Bone. 2013;57: 455–467. 10.1016/j.bone.2013.09.007 24063947 PMC3938967

[60] Andrews JC, Almeida E, van der Meulen MC, Alwood JS, Lee C, Liu Y, et al Nanoscale X-Ray Microscopic Imaging of Mammalian Mineralized Tissue. Microsc Microanal. 2010;16: 327–336. 10.1017/S1431927610000231 20374681 PMC2873966

[61] Bazin D, Carpentier X, Traxer O, Thiaudière D, Somogyi A, Reguer S, et al Very first tests on SOLEIL regarding the Zn environment in pathological calcifications made of apatite determined by X-ray absorption spectroscopy. J Synchrotron Radiat. 2008;15: 506–509. 10.1107/S0909049508014556 18728322

[62] Tang Y, Chappell HF, Dove MT, Reeder RJ, Lee YJ. Zinc incorporation into hydroxylapatite. Biomaterials. 2009;30: 2864–2872. 10.1016/j.biomaterials.2009.01.043 19217156

[63] Weinmann JP. Bone formation and bone resorption. Oral Surg Oral Med Oral Pathol. 1955;8: 1074–1078. 13266337 10.1016/0030-4220(55)90058-x

[64] Furseth R, Johansen E. A microradiographic comparison of sound and carious human dental cementum. Arch Oral Biol.1968;13: 1197–IN13. 10.1016/0003-9969(68)90075-7 4885781

[65] Zander HA, Hurzeler B. Continuous Cementum Apposition. J Dent Res. 1958;37: 1035–1044. 10.1177/00220345580370060301 13611117

[66] Ho SP, Senkyrikova P, Marshall GW, Yun W, Wang Y, Karan K, et al Structure, chemical composition and mechanical properties of coronal cementum in human deciduous molars. Dent Mater. 2009;25: 1195–1204. 10.1016/j.dental.2009.04.005 19464049 PMC2782750

[67] Jang AT, Lin JD, Choi RM, Choi EM, Seto ML, Ryder MI, et al Adaptive properties of human cementum and cementum dentin junction with age. J Mech Behav Biomed Mater. 2014;39C: 184–196. 10.1016/j.jmbbm.2014.07.015 25133753 PMC4265544

[68] Smith AJ, Cassidy N, Perry H, Bègue-Kirn C, Ruch J-V, Lesot H. Reactionary dentinogenesis. Int J Dev Biol. 1995;39: 273–280. 7626417

[69] Schüpbach P, Lutz F, Guggenheim B. Human Root Caries: Histopathology of Arrested Lesions. Caries Res. 1992;26: 153–164. 10.1159/000261436 1628289

[70] Arola D, Reprogel RK. Effects of aging on the mechanical behavior of human dentin. Biomaterials. 2005;26: 4051–4061. 10.1016/j.biomaterials.2004.10.029 15626451

[71] Kinney JH, Balooch M, Haupt DL, Marshall SJ, Marshall GW. Mineral Distribution and Dimensional Changes in Human Dentin during Demineralization. J Dent Res. 1995;74: 1179–1184. 10.1177/00220345950740050601 7790595

[72] Kinney JH, Nalla RK, Pople JA, Breunig TM, Ritchie RO. Age-related transparent root dentin: mineral concentration, crystallite size, and mechanical properties. Biomaterials. 2005;26: 3363–3376. 10.1016/j.biomaterials.2004.09.004 15603832

[73] Omae M, Shinnou Y, Tanaka K, Abo T, Nakata T, Suzuki K, et al XPS analysis of the dentin irradiated by Er: YAG laser. Dent Mater J. 2009;28: 471–476. 19721285 10.4012/dmj.28.471

[74] Zheng L, Hilton JF, Habelitz S, Marshall SJ, Marshall GW. Dentin caries activity status related to hardness and elasticity. Eur J Oral Sci. 2003;111: 243–252. 12786956 10.1034/j.1600-0722.2003.00038.x

[75] Bjørndal L. Dentin and pulp reactions to caries and operative treatment: biological variables affecting treatment outcome. Endod Top. 2002;2: 10–23.

[76] Linde A, Goldberg M. Dentinogenesis. Crit Rev Oral Biol Med. 1993;4: 679–728. 8292714 10.1177/10454411930040050301

[77] Nalbandian J, Gonzales F, Sognnaes RF. Sclerotic Age Changes in Root Dentin of Human Teeth as Observed by Optical, Electron, and X-Ray Microscopy. J Dent Res. 1960;39: 598–607. 10.1177/00220345600390032101 14425932

[78] Ogawa K, Yamashita Y, Ichijo T, Fusayama T. The Ultrastructure and Hardness of the Transparent of Human Carious Dentin. J Dent Res. 1983;62: 7–10. 10.1177/00220345830620011701 6571859

[79] Farah RA, Swain MV, Drummond BK, Cook R, Atieh M. Mineral density of hypomineralised enamel. J Dent. 2010;38: 50–58. 10.1016/j.jdent.2009.09.002 19737596

[80] Nagase H, Suzuki K, Morodomi T, Enghild JJ, Salvesen G. Activation mechanisms of the precursors of matrix metalloproteinases 1, 2 and 3. Matrix Suppl. 1992;1: 237f–244. 1480033

[81] Ogata Y, Enghild JJ, Nagase H. Matrix metalloproteinase 3 (stromelysin) activates the precursor for the human matrix metalloproteinase 9. J Biol Chem. 1992;267: 3581–3584. 1371271

[82] Boskey AL. Matrix Proteins and Mineralization: An Overview. Connect Tissue Res. 1996;35: 357–363. 10.3109/03008209609029212 9084675

[83] Attramadal A, Jonsen J. The content of lead, cadmium, zinc and copper in deciduous and permanent human teeth. Acta Odontol Scand. 1976;34: 127–131. 10.3109/00016357609002559 1067730

[84] Gleń-Haduch E, Szostek K, Głab H. Cribra orbitalia and trace element content in human teeth from Neolithic and Early Bronze Age graves in southern Poland. Am J Phys Anthropol. 1997;103: 201–207. 9209577 10.1002/(SICI)1096-8644(199706)103:2<201::AID-AJPA5>3.0.CO;2-W

[85] Liu K-Z, Hynes A, Man A, Alsagheer A, Singer DL, Scott DA. Increased local matrix metalloproteinase-8 expression in the periodontal connective tissues of smokers with periodontal disease. Biochim Biophys Acta. 2006;1762: 775–780. 10.1016/j.bbadis.2006.05.014 16928431

[86] Sulkala M, Wahlgren J, Larmas M, Sorsa T, Teronen O, Salo T, et al The Effects of MMP Inhibitors on Human Salivary MMP Activity and Caries Progression in Rats. J Dent Res. 2001;80: 1545–1549. 10.1177/00220345010800061301 11499510

[87] Nemeth JA, Yousif R, Herzog M, Che M, Upadhyay J, Shekarriz B, et al Matrix metalloproteinase activity, bone matrix turnover, and tumor cell proliferation in prostate cancer bone metastasis. J Natl Cancer Inst. 2002;94: 17–25. 11773278 10.1093/jnci/94.1.17

[88] Kukacka J, Prusa R, Kotaska K, Pelouch V. Matrix metalloproteinases and their function in myocardium. Biomed Pap. 2005;149: 225–236. 10.5507/bp.2005.031 16601761

[89] Moon PC, Weaver J, Brooks CN. Review of matrix metalloproteinases’ effect on the hybrid dentin bond layer stability and chlorhexidine clinical use to prevent bond failure. Open Dent J. 2010;4: 147 10.2174/1874210601004010147 21339893 PMC3040992

