# Supplementary figures and images for: Mineral Density Volume Gradients in Normal and Diseased Human Tissues
Source: PLoS One. 2015 Apr 9;10(4):e0121611. doi: 10.1371/journal.pone.0121611 (PMC4391782; doi:10.1371/journal.pone.0121611)

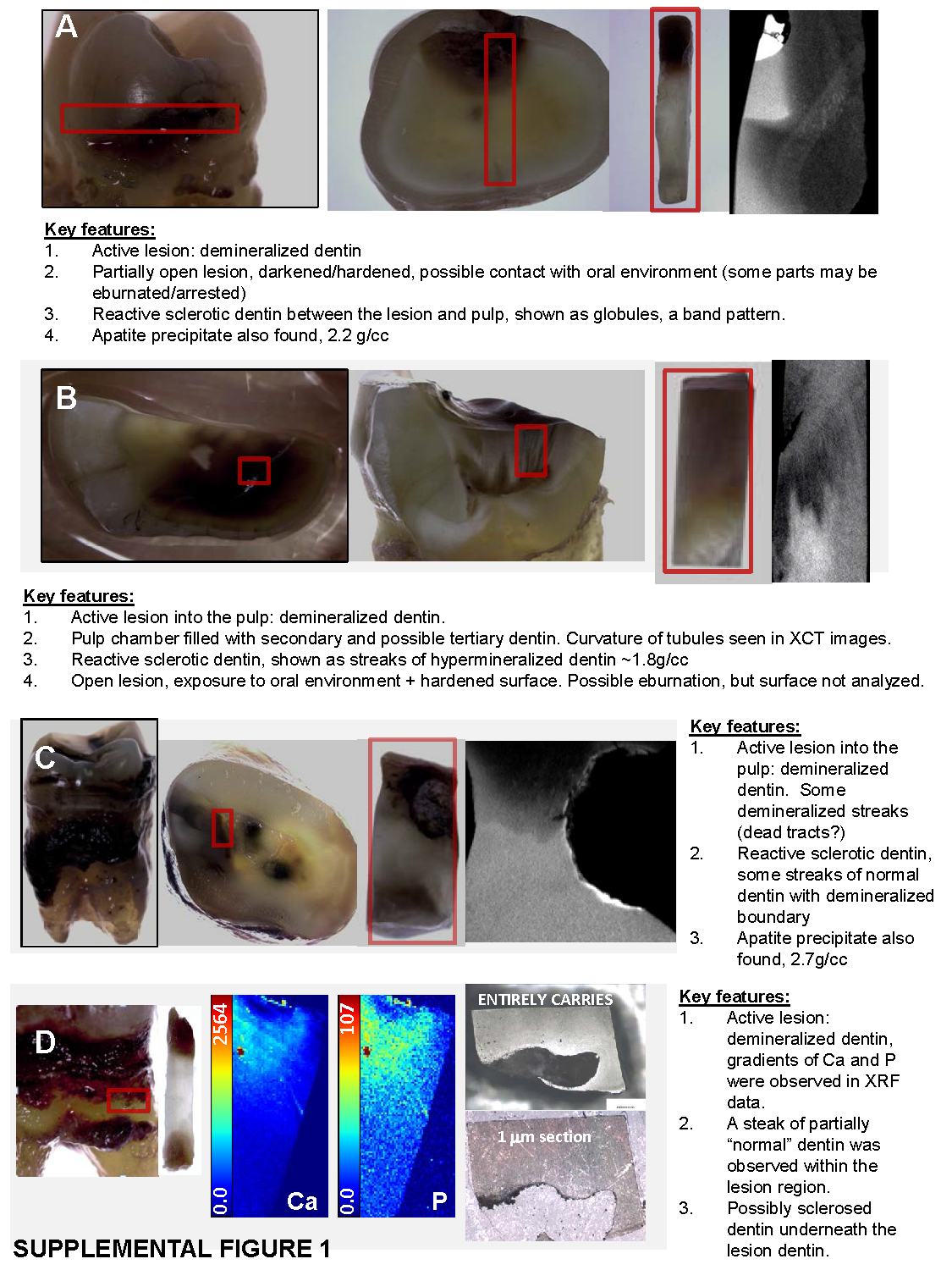

Supplement: S1 Fig — a) carious dentin with active lesion and a dentin beam sectioned from coronal portion of a tooth has revealed sclerotic dentin and hypermineralized zone; b) sclerotic dentin is highlighted by its unique streaking pattern in agreement with observations made by others (Schüpbach 1992); c) periodontally affected tooth with various mineralized zones; d) a carious dentin beam used for XRF imaging that illustrates a hypomineralized zone in dentin towards the lesion site. XRF images of a different dentin slice (5 μm thick) showing Ca and P area maps along with light microscope images of a block dentin specimen affected with caries. (TIF) [file pone.0121611.s001.tif]
