# Supplementary material for: Mineral Density Volume Gradients in Normal and Diseased Human Tissues
Source: PLoS One. 2015 Apr 9;10(4):e0121611. doi: 10.1371/journal.pone.0121611 (PMC4391782; doi:10.1371/journal.pone.0121611)
Supplement: S1 Supplemental Information — (DOCX) [file pone.0121611.s005.docx]

**SUPPLEMENTAL INFORAMTION**

**CT scaling**

CT scaling is performed by acquiring image data that includes both air and a water phantom, and sampling a region of interest (ROI). The mean CT numbers for both water and air are input and saved into the µCT. XMController software, for which all future scans can be scaled to assuming the scan was done at the same experimental parameters (i.e. voltage, magnification, source filter, and beam hardening coefficient). The actual CT scaling on a tomogram is done in the XMReconstructor software, which embeds CT scaling as a feature and allows the user to simply click and apply. After reconstruction is complete, the scan file is said to be CT scaled, and the image data contains a 3D scalar field of HU values.

**Digital Segmentation**

In Fig. 1B and Fig. 2A, volumes of different mineral densities have been represented within a diseased specimen, and the reconstructed volumes and mineral gradients were identified by inputting the CT-scaled reconstructed data for that particular specimen into Avizo® Fire 7 for mineral density segmentation. Steps involved generating a 2D histogram of the gradient magnitude, shown in Fig. 1D with a sample histogram of healthy dentin and healthy enamel. The gradient magnitude at a pixel, computed using standard methods, is defined as the measure of how quickly the intensity changes in the direction of greatest change [16]. Regions are then drawn on the 2D histogram (white boxes, Fig. 1D) representing the HU ranges where segmentation should be performed, and the watershed algorithm is then applied [17,18]. A file is generated after the algorithm is complete, and this is used to binarize each mineral density volume using the quantification tools and thresholding function (in Avizo® Fire 7), which separates and defines the new regions. Once all regions have been defined, the segmentation is overlaid on the original data, and the result is output as spreadsheet file containing columns with the mean HU values of each segmented region. A file is generated after the algorithm is complete, and this is used to binarize each mineral density volume using the quantification tools and thresholding function (in Avizo® Fire 7), which separates and defines the new regions. Once all regions have been defined, the segmentation is overlaid on the original data, and the result is output as spreadsheet file containing columns with the mean HU values of each segmented region. The supplement section further outlines the details.

Furthermore, the process of segmentation is an iterative method where the initial values are identified by their known HU range. The initial HU range for each mineral region can be extracted by thresholding the data from the reconstructed image using a 2D histogram as previously described (Fig. 1D). Hence, the 2D histogram of an image is a plot of intensity in mean HU of each pixel vs. the gradient magnitude or number of pixels representing those intensity values. The number is calculated from the difference in intensities which quantifies how fast its neighbor pixels are changing in intensity. If a pixel is at the intensity edge of a region and its neighbors are either of a significantly higher or of a lower intensity, then the difference is large. If a pixel is in the middle of a uniform region, then its neighboring pixel intensities do not change significantly and as such the number on the Y axis will be smaller. On the 2D histogram, the initial mineral zones are manually selected by drawing in a “seed” region as shown in Fig. 1D (white boxes). The seed region, i.e. the initial range must fall within the HU range for a tissue; but need not span the entire HU range. The watershed algorithm fully segments all mineralized regions in the specimen using the chosen initial range [17,18]. This algorithm is used for separating the different mineral volumes in grayscale images according to their HU intensity, and each mineralized region is iteratively “grown” from the initial seed value. Then through a delimiting process, the HU intensity of neighboring pixels are compared to the mean intensity of the region, and the boundary of each region is defined by those pixels with the highest difference. For images where the HU intensities are cleanly separated for the different mineral regions, slight changes in the dimensions of the seed box (used to calculate the initial value) will not translate into statistically significant differences in resulting mineral density calculations.

The clusters of lowest gradient magnitude (Y-axis, Fig 1D) are chosen as seeds, areas representing the spatial center (or foci) of each homogenous intensity region within a tissue, while the X dimension of the box is the HU value of the tissue. Furthermore, the Y dimension indicates where a minimum change in visible intensity within a tissue exists, ensuring that it does not expand into a visibly different region; it is the gradient magnitude and is defined based on the change in intensity between two adjoining pixels. For example, those shown within boxes are gradients with lowest magnitude as they represent constant mineral density within a tissue. Hence, they appear as bright regions with a value closer to 1 (grayscale color map, Fig. 1D) indicating homogenous material. The actual computation of the colormap in Avizo is simply a conversion from the scalar field of a data set into a label field with linear mapping of data values to a subset of indices in grayscale values (Avizo 7 Reference Guide 2012). This region can be assumed as the seed. Similar seeds can be picked for other phases within a material of interest. Following an iterative process using the seeds as initial values for the watershed algorithm, subsequent zones between different phases are further identified. In the arches shown in Figure 1D, are sub-arcs with intense banding (white arrows). These arches represent the interface between materials where the width of the arch is indicative of intensity roughness at the surface of that material. Note that intensity roughness can be higher for two phases with a jagged junction compared to a straight junction. However, the size of the arch is indicative of the spread of the differential in intensity values. A larger arch indicates a higher differential between two phases, compared to a smaller arch.
